# Dilated superior ophthalmic vein: systemic associations

**DOI:** 10.1007/s10792-023-02782-3

**Published:** 2023-07-01

**Authors:** Khizar Rana, Valerie Juniat, James Slattery, Sandy Patel, Dinesh Selva

**Affiliations:** 1 Department of Ophthalmology and Visual Sciences, University of Adelaide, North Terrace, 5000 Australia; 2 South Australian Institute of Ophthalmology, Royal Adelaide Hospital, Port Road, Adelaide, 5000 Australia; 3 Department of Medical Imaging, Royal Adelaide Hospital, Port Road, Adelaide, 5000 Australia

**Keywords:** Dilated Superior ophthalmic vein, Computed tomography, Magnetic resonance imaging, Orbit

## Abstract

**Purpose:**

To review systemic associations of patients with dilated superior ophthalmic veins (SOV) in the absence of orbital, cavernous sinus, or neurological disease.

**Methods:**

Retrospective review of patients who had dilated SOVs with a diameter of ≥ 5.0 mm. Patients with a dilated SOV secondary to orbital, cavernous sinus or neurological disease were excluded. Patient demographics, past medical history, and SOV diameters on initial and follow up scans were collected. The maximum diameter of the SOV was taken perpendicular to the long axis of the SOV.

**Results:**

Nine cases were identified. Patients ranged in age from 58 to 89 years and six out of nine were female. The dilated SOV involved both eyes in two cases, left eye in five cases and right eye in two cases. Three patients had dilated SOV likely secondary to raised venous pressures from decompensated right heart failure (*n* = 1), pericardial effusion (*n* = 1) and left ventricle dysfunction secondary to a myocardial infarction (*n* = 1). Five patients had a significant history of previous ischaemic heart or peripheral vascular disease. Two patients had risk factors for venous clotting disease whilst one patient had a history of giant cell arteritis and vertebral artery dissection.

**Conclusion:**

A dilated SOV may raise concern for life threatening conditions such as a carotid cavernous fistula and may prompt additional investigations. A dilated SOV may be reversible and secondary to raised venous pressures due to cardiac failure. Other cases may be seen in patients with significant cardiovascular risk factors, possibly due to changes in vasculature.

## Introduction

The orbit is a window to systemic disease [1]. A range of vascular, neoplastic and autoimmune diseases may have orbital manifestations. Likewise, a dilated superior ophthalmic vein (SOV) can occur secondary to an array of conditions in the brain or orbit including vascular diseases e.g., carotid-cavernous fistula, venous thrombosis; inflammatory conditions e.g., idiopathic orbital inflammation and thyroid eye disease; and may be an early indicator of raised intracranial pressure [2–4].

In the absence of any underlying pathology, the mean diameter of the SOV is approximately two millimetres [5–9]. The authors herein review cases of dilated SOVs in the absence of orbital, cavernous sinus or neurological disease.

## Methods

### Patient population

We conducted a retrospective review of asymptomatic patients who were noted to have a dilated superior ophthalmic vein with a diameter of $\ge$5.0 mm on computed tomography (CT) or magnetic resonance imaging (MRI) of the orbits. Patients with a dilated SOV secondary to orbital or cavernous sinus disease were excluded, as were patients with signs or symptoms of raised intracranial pressure. Patient demographics, past medical history, presenting complaint, relevant transthoracic echocardiogram (TTE) findings, indication for neuroimaging, and the SOV diameters were recorded. The study was approved by the Central Adelaide Local Health Network ethics committee and adhered to the principles of the Declaration of Helsinki.

### Imaging

**Fig. 1:**
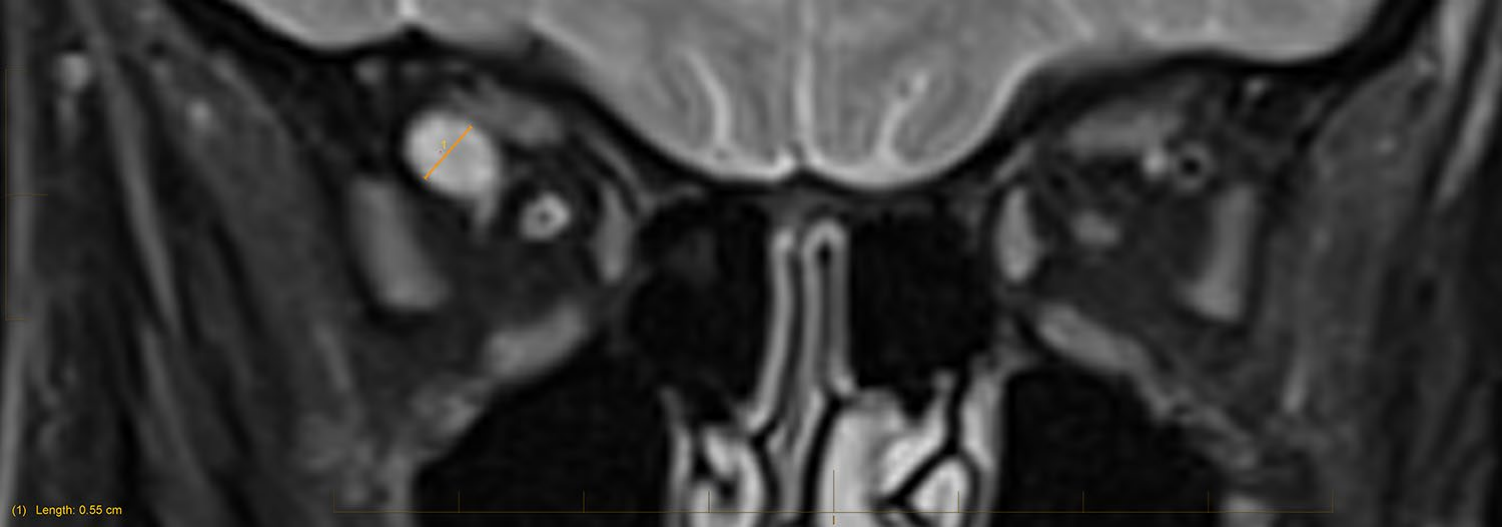
The Superior ophthalmic vein measurement was taken perpendicular to the long axis of the SOV on T2-fat suppressed coronal sequences

All patients were evaluated using Magnetom 3 T Skyra scanner (Siemens, Germany) or SOMATOM Force CT (Siemens AG, Germany). The maximum diameter of the SOV was taken perpendicular to the long axis of the SOV on T2 coronal sequences or coronal CT scan (Fig. 1) [2]. T2 coronal sequences are used in line with previous reports [2, 5]. In patients who did not have scans following the dilated scan result, the SOV sizes from any previous scans were recorded. All measurements were performed on high resolution picture archiving and communication system (PACS) under the supervision of a consultant neuroradiologist (SP).

The normal diameter of the SOV is approximately 2 mm [5, 6, 8, 10]. We used a value of 5 mm to define a dilated SOV to ensure we captured cases that would fall outside of the normal two standard deviation variation and would otherwise raise suspicion for a pathological underlying cause [6, 8].

## Results

We identified nine patients with dilated SOVs. Six (67%) patients were female, and the mean age was 72 years (range 58–89). Patient demographics are provided in Table 1. The maximum diameters of the SOV on the initial and other scans are provided in Table 2. Five patients had follow-up imaging after the dilated SOV scan, whereas neuroimaging from before the dilated SOV scan was evaluated in three other cases. Small fluctuations within the size of the SOV may be attributed to dynamic changes in venous pressures. Patients who had follow up scans after the dilated SOV scan did not show a significant increase in the size of the SOV and remained asymptomatic without any visual complications.

**Table 1:** Clinical profile of patients with dilated superior ophthalmic vein

Case	Age	Sex	Past medical history	Presenting complaint	Initial indication for neuroimaging	Transthoracic echocardiogram
1	68	F	T2DM, IHD with PCI, Hypercholesterolemia, MV repair	Decompensated right heart failure	Exclude superior vena cava obstruction	Dilated right heart with reduced right ventricular free wall contraction
2	74	M	IHD with CABG × 3, CKD, CLL	Fall	Exclude intracranial bleed	Moderate to severe global systolic dysfunction
3	59	F	Factor V leiden, AF, Psoriasis	Left upper neuropathy	Investigate left upper neuropathy cause	NA
4	80	F	Inferior STEMI, HTN, Colorectal cancer, SCC	Infraorbital paraesthesia	Exclude perineural invasion	Basal inferior hypokinesis
5	75	M	BRVO left and right	Visual obscuration	Exclude vasculitis or ischaemia	NA
6	89	F	Angina, HTN, Hypercholesterolemia	Anterior Myocardial infarction	Code Stroke following PCI	Moderate systolic dysfunction. Septal, mid to distal anterior and apical inferolateral akinesis. EF 41%
7	66	F	Spondylarthritis, HLA B27 + ve	Bilateral PE and Pericardial effusion due to metastatic breast cancer	Exclude brain metastases	Pericardial effusion with mildly dilated right ventricle with mid wall akinesis
8	58	M	PVD with stent, Popliteal aneurysms, Hypercholesterolemia, Smoker	Gait ataxia	Code stroke	NA
9	82	F	Biopsy Proven GCA, HTN, SVT	GCA relapse, incidental Vertebral artery dissection	4th nerve palsy, headaches. Evidence of GCA?	NA

*T2DM* type 2 diabetes mellitus; *IHD* ischaemic heart disease; *PCI* percutaneous coronary intervention; *MV* mitral valve; *CKD* chronic kidney disease; *CABG* coronary artery bypass graft; *SCC* squamous cell carcinoma; *CLL* chronic lymphocytic leukemia; *AF* atrial fibrillation; *HTN* Hypertension; *PE* pulmonary embolism; *PVD* peripheral vascular disease; *SVT* supraventricular tachycardia; *GCA* giant cell arteritis; *BRVO* branch retinal vein occlusion; *EF* ejection fraction; *NA* not available

**Table 2:** Superior ophthalmic vein measurements

Scan with dilated SOV			Other neuroimaging				Interval change between most recent scan and earlier scans	
Case	Right SOV diameter (mm)	Left SOV diameter (mm)	Prior to or after dilated SOV scan	Time difference between scans (years)	Right SOV diameter (mm)	Left SOV diameter (mm)	Difference in right SOV	Difference in left SOV
1	9.4	12.5	After	2.4	1.5	1.5	− 7.9	− 11
2	2.6	6.8	After	0.4	4.0	4.5	+ 1.4	− 2.3
3	3.0	5.1	After	1.2	3.5	5.5	+ 0.5	+ 0.4
4	5.5	2.6	After	0.6	4.1	3.7	− 1.4	+ 1.1
5	3.1	6.8	Before	0.9	1.2	1.4	+ 1.9	+ 5.4
6	10.5	10	Before	5.3	4.6	5.9	+ 5.9	+ 4.1
7	6.3	4.1	After	1.7	0.8	0.9	− 5.5	− 3.2
8	4.2	5.2	Before	0.03	1.8	1	+ 2.4	+ 4.2
9	3.5	7.2	NA					

Five patients had a history of significant cardiovascular disease. Four patients had previous or current presentations with myocardial infarction requiring revascularisation (coronary artery bypass graft [CABG] or percutaneous coronary intervention [PCI]). One patient had a history of peripheral vascular disease requiring a stent.

**Fig. 2:**
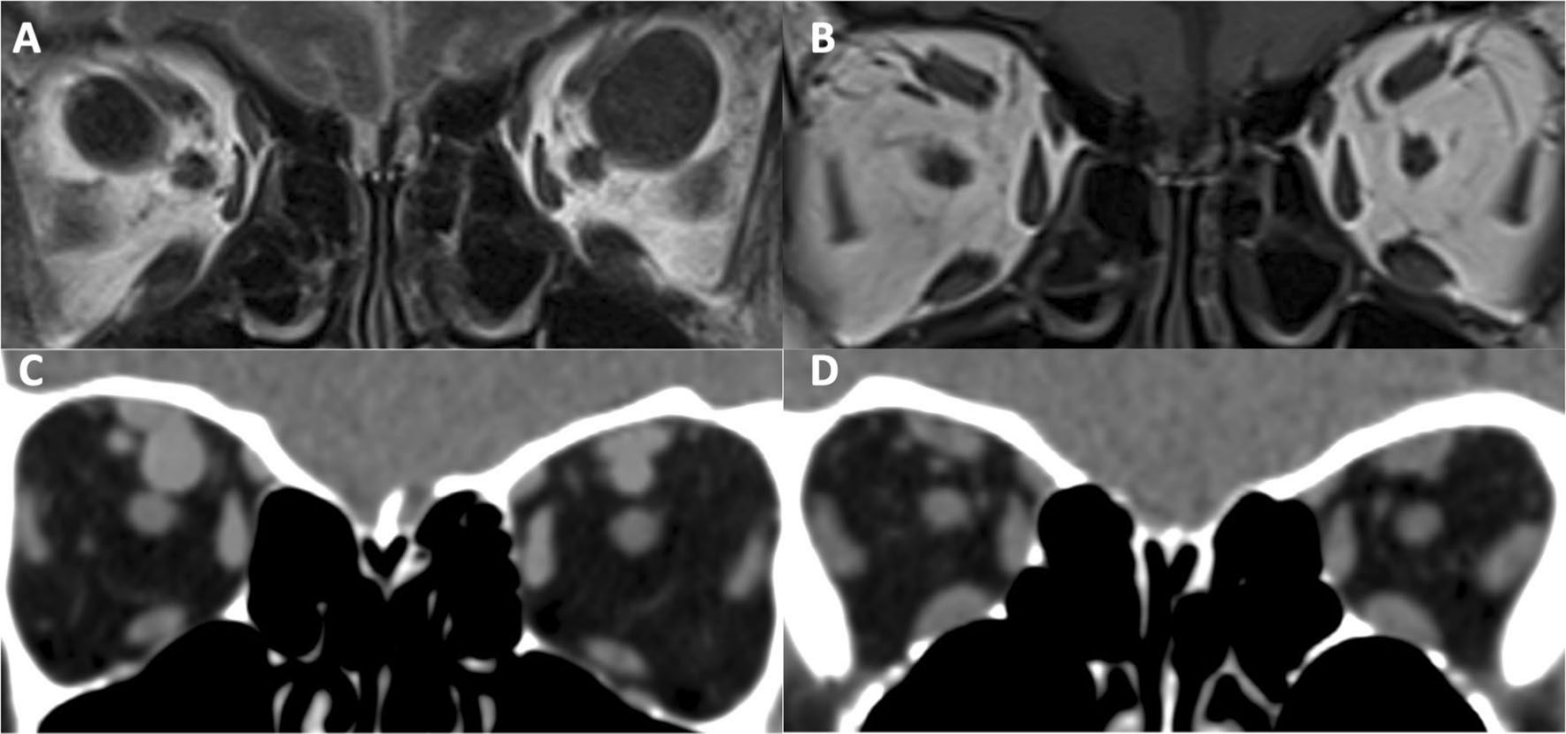
Initial T1-Weighted coronal MRI (**A**) from Case 1 showing bilateral SOV dilatation. This patient presented with decompensated right heart failure. A follow up scan (**B**) two years later revealed normal SOVs. Initial coronal CT scan (**C**) showing bilateral SOV dilatation in Case 7. This patient had a pericardial effusion secondary to breast cancer. A follow up CT scan (**D**) showed normal SOVs

Three cases (1, 6, 7) had evidence of ‘reversible’ SOV dilatation likely secondary to raised venous pressures. Case 1 presented with decompensated right heart failure with ascites, peripheral oedema, and facial oedema. Case 7 presented with bilateral pulmonary emboli, in the context of metastatic breast cancer complicated by a malignant pericardial effusion with impending tamponade. They had bilateral SOV dilatation which resolved to within normal limits on follow-up scans (Fig. 2). Similarly, case 6 presented with a myocardial infarction with the TTE showing evidence of left ventricle dysfunction and had an elevated B-type natriuretic peptide (10,784). A review of this patient’s imaging from five years prior revealed that he had pre-existing, although less severe SOV dilatation (left 4.6 mm, right 5.9 mm) that was likely exacerbated by his current presentation of left ventricular dysfunction.

Two cases (cases 3, 5) had significant venous clotting risk factors. Case 3 had a history of Factor V Leiden, whilst Case 5 had a history of bilateral branch retinal vein occlusion (BRVO).

## Discussion

This is the first study looking at dilated SOVs in the absence of orbital, cavernous sinus or neurological disease. All the patients were asymptomatic from an ophthalmic perspective and patients with follow up scans after the dilated scan did not show significant progression in this size of the SOV. Analysis of these cases may offer some insights into the potential pathophysiology of dilated SOVs and may reveal an association between cardiovascular disease and dilated SOVs.

**Fig. 3:**
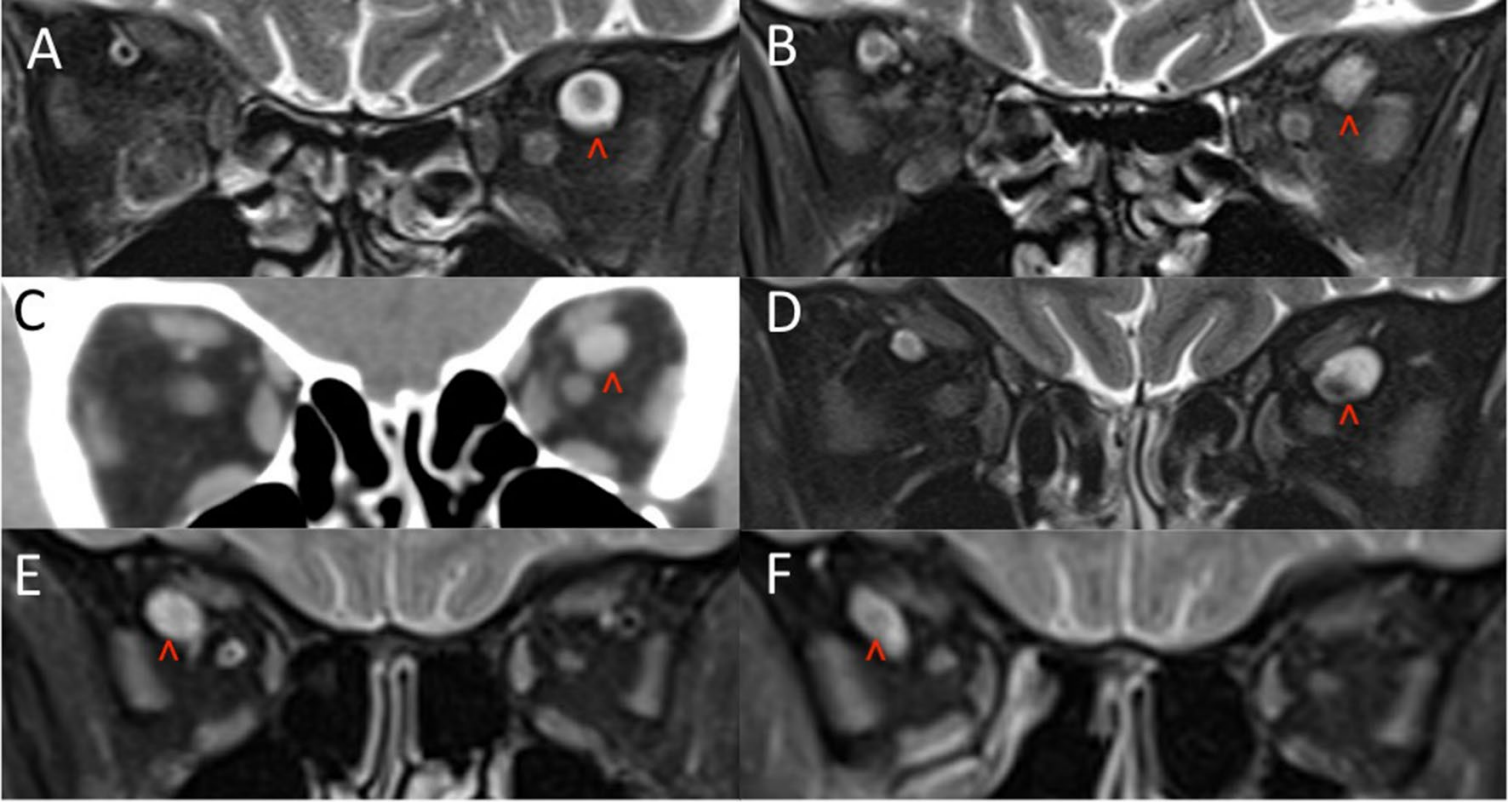
Initial (**A**) and follow up (**B**) coronal T2 fat suppressed scans in case 2 showing a left dilated SOV. Initial CT (**C**) and follow up coronal MRI (**D**) showing a left dilated SOV in case 3. Case 4 shows a right dilated SOV on initial (**E**) and follow up (**F**) scans

Elevated venous pressures may explain the dilated SOVs in three of our patients. These patients presented with decompensated right heart failure (case 1), acute anterior myocardial infarction (case 6) and pericardial effusion (case 7). All of these patients had bilateral SOV dilatations. All of these patients had symptomatic and severe cardiac failure. Case 1 presented with decompensated right heart failure with peripheral and facial oedema such that imaging was performed to exclude superior vena cava obstruction. Case 6 had an acute anterior myocardial infarction. Case 7 had a pericardial effusion, which two days after the CT brain, was urgently drained via pericardiocentesis as it caused a cardiac tamponade. Cases 1 and 7 had follow up scans which showed resolution of the SOV dilatation, likely due to treatment of the underlying cause (decompensated heart failure, pericardial effusion). The SOV dilatation was likely transient and in response to elevated venous pressures secondary to acute cardiac disease. Following stabilisation of the acute disease, the SOV sizes reduced. The other cases where unilateral dilatation was seen may have a different underlying pathophysiology (Fig. 3).

Five patients had a significant history of ischaemic heart disease or peripheral vascular disease. It has been suggested that both arterial and venous dilating disease may arise from a common vascular wall pathology [11]. Risk factors for arterial aneurysms (e.g. hypertension, hypercholesterolemia, male sex, increasing age, smoking) may also play a role in the vascular wall degeneration and dilatation of venous vessels [12]. Increased nitric oxide stimulation plays a role in the pathogenesis of arterial dilatations such as abdominal aortic aneurysms, and has also been linked to the development of venous dilatations including varicose veins [13]. (Fig. 3).

Local factors may also play a role in the pathophysiology of dilated SOVs. Valvular incompetence is implicated in the development of venous dilatations in the lower limbs [13]. Although the SOV has traditionally been thought to be valveless [6, 14, 15], a cadaveric study analysing twelve specimens of the SOV has found evidence of valves in four of the twelve specimens [16]. Valvular incompetence may have a role to play in the development of dilated SOVs, similar to other areas of the body. Additionally, valvular incompetence is usually asymmetric, potentially explaining the unilateral dilatation in some of our cases.

Two cases (cases 3, 5) had significant venous clotting risk factors. Case 3 had a history of Factor V Leiden, whilst Case 5 had a history of bilateral BRVO. Patients with procoagulant risk factors such as active cancer or Factor V leiden carriers have been reported to have asymptomatic thromboses in other areas of the body [17, 18]. It may be the case that our two patients may have had asymptomatic thromboses of the superior ophthalmic vein, that may have led to the dilated SOVs. There was however no evidence of an acute thrombus.

This study has some limitations. This is a retrospective study on a small sample size, due to the rarity of this condition. Further cases from additional centres may help to clarify the potential role of cardiovascular disease in the development of a dilated SOV, in patients who are otherwise asymptomatic from an ocular perspective.

Ophthalmologists may be consulted to give an opinion on incidental findings such as SOV dilatation. SOV dilatation may simply reflect elevated venous pressures secondary to cardiac failure and may self-resolve with treatment of the underlying condition. Other cases of dilated SOV may be seen in patients with significant ischaemic heart disease, peripheral vascular disease, or venous clotting history. These patients may be safely observed as long as they remain asymptomatic.

## References

[1] McNab AA (2018). The 2017 doyne lecture: the orbit as a window to systemic disease. Eye.

[2] Adam CR, Shields CL, Gutman J, Kim HJ, Hayek B, Shore JW, Braunstein A, Levin F, Winn BJ, Vrcek I, Mancini R, Linden C, Choe C, Gonzalez M, Altschul D, Ortega-Gutierrez S, Paramasivam S, Fifi JT, Berenstein A, Durairaj V, Shinder R (2018). Dilated Superior Ophthalmic Vein: Clinical and Radiographic Features of 113 Cases. Ophthalmic Plast Reconstr Surg.

[3] Peyster RG, Savino PJ, Hoover ED, Schatz NJ (1984). Differential diagnosis of the enlarged superior ophthalmic vein. J Comput Assist Tomogr.

[4] Flynn E, Suri H, Stevens J, Kataria L (2018). Bilateral superior ophthalmic vein dilatation as an early indicator of increased cranial pressure (P4.007). Neurology.

[5] Tsutsumi S, Nakamura M, Tabuchi T, Yasumoto Y (2015). The superior ophthalmic vein: delineation with high-resolution magnetic resonance imaging. Surg Radiol Anat.

[6] Brightbill TC, Martin SB, Bracer R (2001). The diagnostic significance of large superior ophthalmic veins in patients with normal and increased intracranial pressure: CT and MR evaluation. Neuroophthalmology.

[7] Ahmadi J, Teal JS, Segall HD, Zee CS, Han JS, Becker TS (1983). Computed tomography of carotid-cavernous fistula. AJNR Am J Neuroradiol.

[8] Ozgen A, Aydingöz U (2000). Normative measurements of orbital structures using MRI. J Comput Assist Tomogr.

[9] Reis CV, Gonzalez FL, Zabramski JM, Hassan A, Deshmukh P, Albuquerque FC, Preul MC (2009). Anatomy of the superior ophthalmic vein approach for direct endovascular access to vascular lesions of the orbit and cavernous sinus. Neurosurgery.

[10] Rana K, Juniat V, Rayan A, Patel S, Selva D (2022). Normative measurements of orbital structures by magnetic resonance imaging. Int Ophthalmol.

[11] Yetkin E, Ozturk S (2018). Dilating Vascular Diseases: Pathophysiology and Clinical Aspects. Int J Vasc Med.

[12] Androulakis AE, Katsaros AA, Kartalis AN, Stougiannos PN, Andrikopoulos GK, Triantafyllidi EI, Pantazis AA, Stefanadis CI, Kallikazaros IE (2004). Varicose veins are common in patients with coronary artery ectasia. Just a coincidence or a systemic deficit of the vascular wall?. Eur J Vasc Endovasc Surg.

[13] Jacobs BN, Andraska EA, Obi AT, Wakefield TW (2017). Pathophysiology of varicose veins. J Vasc Surg Venous Lymphat Disord.

[14] Servo A (1982). Visualization of the superior ophthalmic vein on carotid angiography. Neuroradiology.

[15] Derang J, Ying H, Long Y, Reifa S, Qiming W, Yimu F, Guoxiang S, Shimin C, Lihua X, Shi W, Zunhua C (1999). Treatment of carotid-cavernous sinus fistulas retrograde via the superior ophthalmic vein (SOV). Surg Neurol.

[16] Zhang J, Stringer MD (2010). Ophthalmic and facial veins are not valveless. Clin Exp Ophthalmol.

[17] Gary T, Belaj K, Steidl K, Pichler M, Eisner F, Stöger H, Hafner F, Froehlich H, Samonigg H, Pilger E, Brodmann M (2012). Asymptomatic deep vein thrombosis and superficial vein thrombosis in ambulatory cancer patients: impact on short-term survival. Br J Cancer.

[18] Simioni P, Tormene D, Prandoni P, Zerbinati P, Gavasso S, Cefalo P, Girolami A (2002). Incidence of venous thromboembolism in asymptomatic family members who are carriers of factor V Leiden: a prospective cohort study. Blood.

